# Gas-Phase Fragmentation Reactions of Protonated Cystine using High-Resolution Tandem Mass Spectrometry

**DOI:** 10.3390/molecules24040747

**Published:** 2019-02-19

**Authors:** Pengwei Zhang, Wan Chan, Irene L. Ang, Rui Wei, Melody M. T. Lam, Kate M. K. Lei, Terence C. W. Poon

**Affiliations:** 1 Pilot Laboratory, Institute of Translational Medicine, Faculty of Health Sciences, University of Macau, Macau, China; yb47620@connect.um.edu.mo (P.Z.); ireneang@um.edu.mo (I.L.A.); yb47623@connect.um.edu.mo (R.W.); katelei@um.edu.mo (K.M.K.L.); 2 Department of Chemistry, Hong Kong University of Science and Technology, Hong Kong, China; chanwan@ust.hk; 3 Proteomics Core, Faculty of Health Sciences, University of Macau, Macau, China; mantinglam@um.edu.mo

**Keywords:** cystine, cysteine, gas-phase fragmentation reaction, high-resolution MS/MS, pseudo MS^3^, isobaric fragment

## Abstract

Cystine is an important biomolecule in living systems. Although collision-induced dissociation (CID)-based tandem mass spectrometry (MS/MS) is commonly applied for identification and quantification of cystine in both biomedical and nutritional studies, gas-phase fragmentation reactions of cystine in CID has remained unclear. This may lead to improper assay design, which may in turn result in inaccurate test results. In the present study, gas-phase fragmentation reactions of protonated cystine in CID were characterized using high-resolution MS/MS and pseudo MS^3^. Fragmentations started from cleavages of disulfide bond (S–S) and carbon–sulfur bond (C–S). When cleaving at the S–S, protonated cysteine was generated as one of the predominant fragmentation products. Minor fragmentations started from the loss of H_2_O + CO and the loss of NH_3_. Our results reveal that the *m/z* 74 fragment ion, which is commonly used as a product ion of the transition (precursor/product ion pair) in selected reaction monitoring (SRM) assay for quantifying cystine, comprises two isobaric fragments originating from different parts of cystine. This indicates the need for careful selection of a stable isotope-labeled cystine molecule as an internal standard for SRM assays. Here, we provide a clear picture of the fragmentation reactions of protonated cystine in CID. It can serve as a useful guidance for designing MS/MS-based assays for cystine testing.

## 1. Introduction

Studies of gas-phase fragmentation reactions of protonated amino acids, particularly in collision-induced fragmentation (CID), are crucial not only for their identification and quantification but also for facilitating the understanding of the fragmentation reactions of its analogous and small peptides [1,2,3,4]. Cystine, an oxidized form of cysteine, is a special dimer of cysteine. Although it is a nonproteinogenic amino acid, cystine plays important roles in a variety of cellular functions and is involved in metabolism pathways [5]. Cystine in urine, blood, or other biological samples has been used as an important biomarker for various pathological conditions, such as inherited metabolic disorders and cystinosis [4,5,6]. Quantification of cystine in leukocytes and urine by CID-based LC–MS/MS is performed in the clinical routine for diagnosis of nephropathic cystinosis [7,8,9,10]. Selected reaction monitoring (SRM) is the mostly reported method for measuring cystine [6,7,8,9,10,11,12,13]. Thus, a good knowledge of the gas-phase fragmentation chemistry of cystine in CID is essential to design MS/MS-based assays for reliable cystine testing. Furthermore, it can help in understanding the fragmentation chemistry of S–S and C–S bond-containing molecules, such as peptides and other biomolecules, which in turn facilitates development of strategies for structure elucidation [14]. 

To the best of our knowledge, MS/MS fragmentation reactions of protonated cystine in CID have only been reported by three research teams [4,14,15,16]. In total, nine fragment ions were reported at *m/z* 74, *m/z* 120, *m/z* 122, *m/z* 152, *m/z* 154, *m/z* 178, *m/z* 195, *m/z* 223, and *m/z* 224. Only two fragment ions at *m/z* 122 and *m/z* 195 were reported by all three research teams. The inconsistency could be due to the use of standard or in-house optimized MS/MS procedures in the three studies. Except the fragment ion at *m/z* 74, chemical identities were assigned to those observed fragmentation products [14,15,16]. It is important to note that, in all these experiments, their CID-based MS/MS platforms only had unit mass resolution, which could not resolve intramolecular isobaric fragment ions. This might have resulted in incorrect assignments of the chemical identities for the observed fragmentation products. Such annotation ambiguity can only be solved using high-resolution tandem mass spectrometry (HR-MS/MS) [17,18]. 

Because only limited information about the fragmentation chemistry of protonated cystine is available, the present study aimed to characterize the gas-phase fragmentation reactions of protonated cystine in CID using HR-MS/MS. Together with pseudo MS^3^ experiments, fragmentation reactions of the protonated cystine was clearly elucidated.

## 2. Results and Discussion

**Figure 1:**
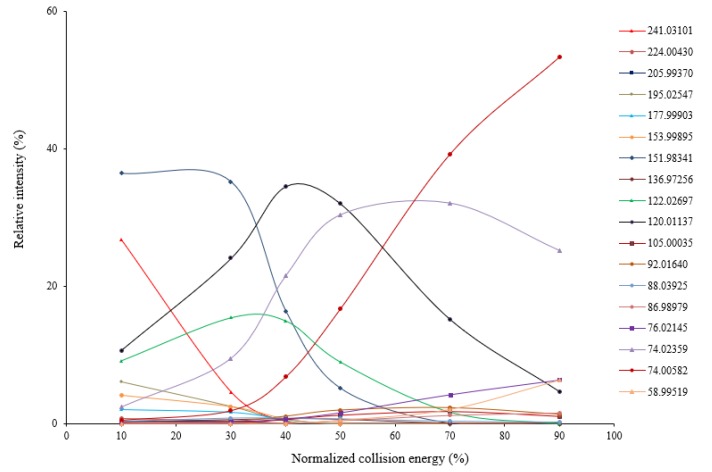
Energy-resolved fragmentation graph of protonated cystine under different collision energies.

Using HR-MSMS operated at various levels of collision energy, gas-phase fragmentation of protonated cystine (*n* = 3) generated 17 reproducible fragment ions for which chemical identities were successfully assigned with a mass tolerance of 5 ppm. Sixteen of the 17 fragment ions had a mass error ≤ 1.5 ppm. Nine of the 17 fragment ions were observed in the previous studies using CID or other fragmentation techniques [4,14,15,16,19,20]. Their assigned chemical identities are summarized in Table 1. Under low collision energy (normalized collision energy, NCE ≤ 30%), the fragment ions at *m/z* 151.98341, *m/z* 122.02697, and *m/z* 120.01137 were the predominant fragmentation products (Figure 1). When the collision energy increased, their relative intensities decreased rapidly, and two isobaric fragment ions at *m/z* 74.02359 and 74.00582 became predominant. Using electron-induced dissociation (EID)-based HR-MS/MS, only the isobaric fragment ion at *m/z* 74.0240 was observed [16]. Relative intensities of other fragment ions were always low, regardless of the collision energy levels. Energy-resolved fragmentation graph of protonated cystine is provided in Figure 1. 

**Figure 2:**
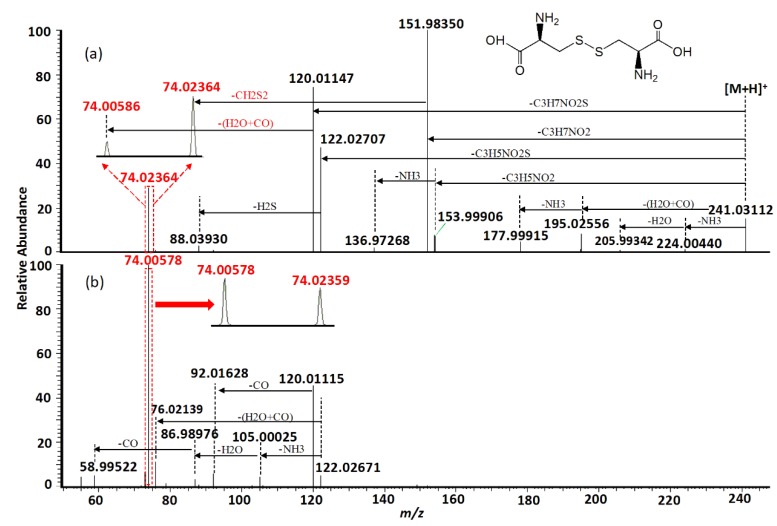
Representative MS/MS spectra of protonated cystine acquired using normalized collision energy (NCE) of (**a**) 30% and (**b**) 70%. Isobaric fragmentations are shown in red.

**Figure 3:**
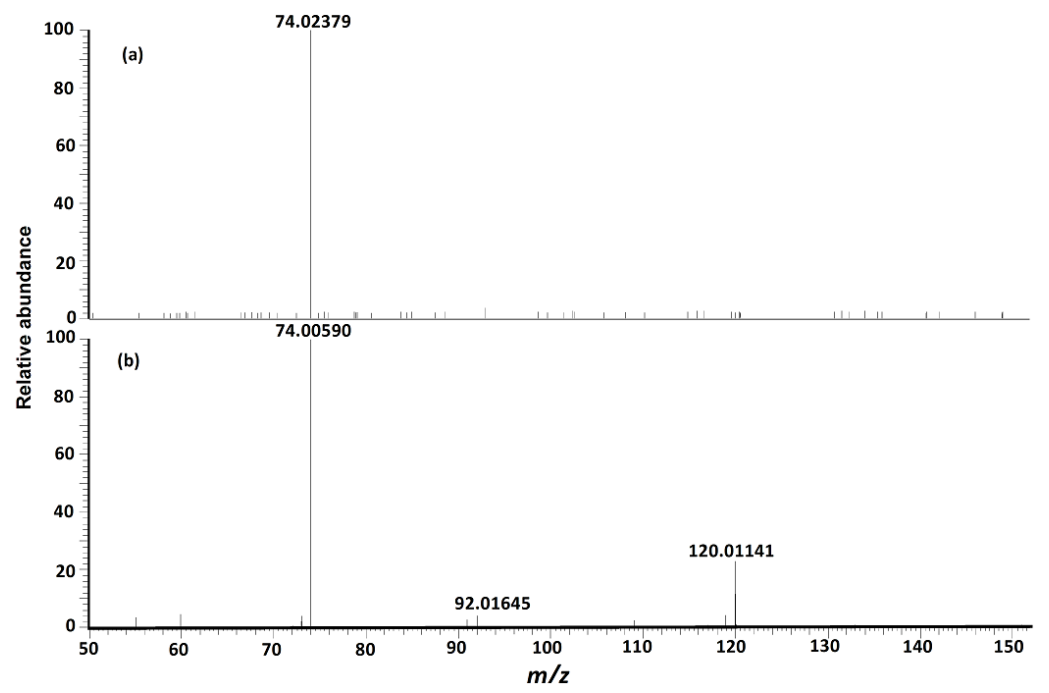
(**a**) Pseudo MS^3^ spectrum of fragment ion at *m/z* 151.98341 from protonated cystine, which supports the proposed fragmentation pathway for formation of fragment at *m/z* 74.02359. (**b**) Pseudo MS^3^ spectrum of the fragment ion at *m/z* 120.01137 from protonated cystine, which supports the proposed fragmentation pathway for formation of fragments at *m/z* 74.00582 and *m/z* 92.01640.

MS/MS fragmentation reactions of protonated cystine mainly involved four major pathways with initial cleavages at the C–S bond and S–S bond (Figure 2a). First, a pair of fragment ions at *m/z* 151.98341 (loss of C_3_H_7_NO_2_, major form) and *m/z* 153.99895 (loss of C_3_H_5_NO_2_, minor form) was formed through the cleavage of C–S bond. The *m/z* 151.98341 fragment ion was fragmented to form the *m/z* 74.02359 fragment ion after the further loss of CH_2_S_2_ through the cleavage of C2–C3 or C2′–C3′ bond. This fragmentation reaction was confirmed by pseudo MS^3^ analysis of the fragment ion at *m/z* 151.98341 (Figure 3a). Second, the cleavage of S–S bond generated two fragment ions at *m/z* 120.01137 and *m/z* 122.02697 by the loss of C_3_H_7_NO_2_S and loss of C_3_H_5_NO_2_S, respectively (Figure 2a). The *m/z* 120.01137 fragment ion was further dissociated to form two fragment ions at *m*/*z* 92.01640 and *m*/*z* 74.00582 by the loss of CO (Figure 2b) and loss of H_2_O + CO (Figure 2b), respectively. These two fragments were also confirmed by pseudo MS^3^ analysis of the fragment ion at *m/z* 120.01137 (Figure 3b). 

With respect to the loss of C_3_H_5_NO_2_S, the *m/z* 122.02697 fragment should be protonated cysteine. This chemical identity was verified by examining its fragmentation products. Under low collision energy, the *m/z* 122.02697 fragment ion was dissociated to form a fragment ion at *m/z* 88.03925 with the loss of H_2_S. Under high collision energy, the *m/z* 122.02697 fragment ion was dissociated to form a fragment ion at *m/z* 76.01245 after the loss of H_2_O + CO, whereas sequential losses of NH_3_, H_2_O, and CO from the *m/z* 122.02697 fragment ion led to the formation of fragment ions at *m/z* 105.00035, *m/z* 86.98979, and *m/z* 58.99519, respectively (Figure 2b). Except for the minor fragment ion at *m/z* 88.03925, all fragmentation products of the *m/z* 122.02697 fragment ion were identical with the previously reported MS/MS fragmentation products of protonated cysteine [21]. 

**Figure 4:**
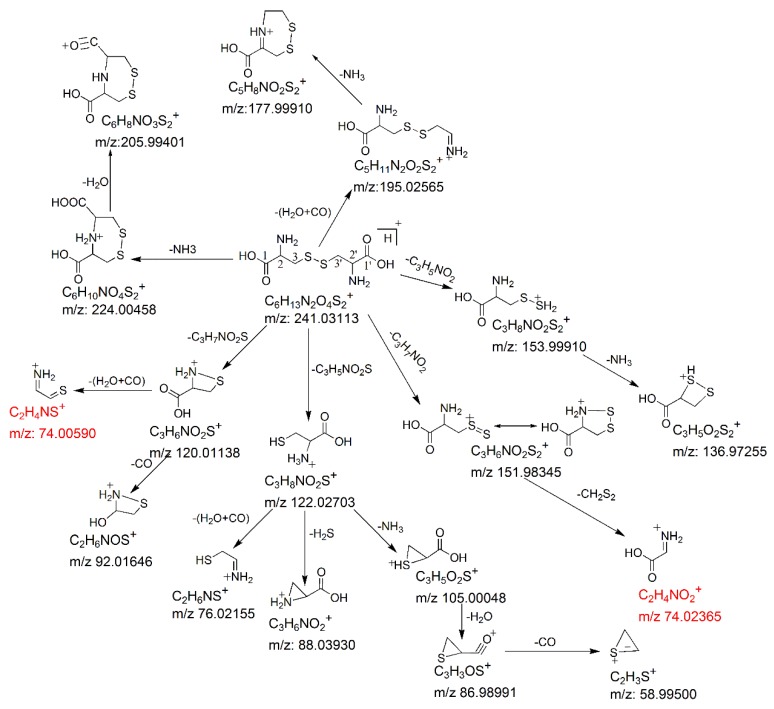
Postulated fragmentation pathways for protonated cystine in CID. The isobaric fragments are shown in red. The *m/z* values shown are the theoretical masses obtained by calculation.

Besides the four major fragmentation pathways, there were two minor pathways for protonated cystine. Under low collision energy, the loss of H_2_O + CO and loss of NH_3_ from protonated cystine resulted in the formation of two fragment ions at *m/z* 195.02547 and *m/z* 224.00430, respectively (Figure 2a), which is similar to the fragmentation of protonated α-amino acids [21]. A further loss of NH_3_ from the *m/z* 195.02547 fragment led to the formation of a fragment ion at *m/z* 177.99903 (Figure 2a). The postulated fragmentation pathways of protonated cystine are presented in Figure 4. 

Cystine plays an important role in biological systems. Its levels in biological samples are commonly measured by SRM. The SRM transition 241→74 [4,10,12,13,22] or 241→152 [6,7,8,9,11,22] is mostly used for cystine quantification to increase sensitivity. Our data showed that the *m/z* 152 fragment ion was predominant when the collision energy was low (Figure 2a). In contrast, the *m/z* 74 fragment ion was predominant when the collision energy was high (Figure 2b). Therefore, the selection between these two fragment ions as the product ion of the SRM transition for maximum sensitivity depended on the collision energy chosen in the SRM assay. Moreover, our data showed that the *m/z* 74 fragment ion comprised two isobaric fragments (i.e., theoretical monoisotopic masses of *m/z* 74.00590 and *m/z* 74.02365), which were originated from different parts of cystine (Figure 4) and had similar intensities (Figure 2b). The *m/z* 74.00590 fragment ion contained carbon atoms at the C2 and C3 positions or at the C2′ and C3′ positions, whereas the *m/z* 74.02365 fragment ion contained carbon atoms at the C1 and C2 positions or at the C1′ and C2′ positions. In SRM assays, stable isotope-labeled cystine is spiked into the samples and used as an internal standard. Some examples of commercially available stable isotope-labeled cystine are cystine-1,1′-^13^C2 (Example 1), cystine-^15^N2 (Example 2), cystine-3,3,3′,3′-d4 (Example 3) and cystine-^15^N2-1,2,3,1′,2′,3′-^13^C6 (Example 4). Corresponding to the pair of *m/z* 74 isobaric fragment ions (i.e., *m/z* 74.00590 and *m/z* 74.02365) from unlabeled cystine, Example 1 will generate two fragment ions at *m/z* 74 and *m/z* 75; Example 2 will generate two fragment ions at *m/z* 75 and *m/z* 75; Example 3 will generate two fragment ions at *m/z* 76 and *m/z* 74; and Example 4 will generate two fragment ions at *m/z* 78 and *m/z* 78. Only Example 2 and Example 4 will generate two isobaric fragment ions, whereas Example 1 and Example 3 will generate two fragment ions of different masses. In Example 1 and Example 3, one of the fragment ions is exactly the same as the corresponding *m/z* 74 fragment ion from unlabeled cystine. If Example 1 or Example 3 is selected as an internal standard in a SRM assay that uses transition 241→74 for measuring signal intensity from endogenous cystine (unlabeled) in a sample, the *m/z* 74 fragment ion from the internal standard will interfere the signal intensity of the *m/z* 74 fragment ion from the endogenous cystine. These simple examples indicate the importance of careful selection of a stable isotope-labeled cystine molecule as an internal standard for SRM assays.

In conclusion, gas-phase fragmentation reactions of protonated cystine in CID were characterized using HR-MS/MS for the first time. Here, we provide a clear picture of the fragmentation reactions of protonated cystine in CID. This can serve as a useful guidance for designing MS/MS-based assays for cystine testing. 

## 3. Materials and Methods 

### 3.1. Materials

l-cystine (purity = 99.9%, pharmaceutical secondary standard, Cat. No. PHR1323, Lot. No. LRAA0826) was obtained from Sigma-Aldrich, St. Louis, MO, USA. LC-MS grade formic acid (FA), water and acetonitrile (ACN) were obtained from Thermo Fisher Scientific, Pierce Chemical, Rockford, IL, USA. l-Cystine stock solution was prepared in MS grade water containing 0.1 M HCl and stored at −80 °C. Before analysis, the stock solution was diluted to 5 μM working solution using ACN/water (1:1).

### 3.2. High-Resolution Tandem Mass Spectrometry (HR-MS/MS)

MS/MS fragmentations by HCD, which is a CID technique specific to the orbitrap mass spectrometer, were conducted on a Q Exactive Hybrid Quadrupole-Orbitrap Mass Spectrometer (Thermo Fisher Scientific, Waltham, MA, USA) equipped with a heated electrospray source. The MS/MS parameters were set as follows: MS/MS resolution, 70,000; automatic gain control (AGC), 5 × 10^5^; injection time, 250 ms; isolation window, 0.4 Da. Ten microliters quantity of the working solution was directly injected to the MS by a UHPLC (UltiMate 3000 RSLCnano System, Thermo Fisher Scientific, Waltham, MA, USA) with an autosampler using an isocratic gradient of 50% ACN containing 0.1% FA at a flow rate of 0.1 mL/min. The ion source parameters were set as follows: spray voltage, 3.0 kV; sheath gas, 25 (arbitrary unit); Aux gas, off; Aux gas heat, off. For pseudo MS^3^ acquisition, an in-source fragment from the parent ion was isolated by the quadrupole (size of isolation window = 0.4 Da) and fragmented in the collision cell. The experiments were conducted in triplicates on different days. Data obtained from the triplicate experiments were used to calculate the mean values of the relative intensity (relative to the total fragment intensity) and *m/z* value of each fragment. Chemical identities were assigned to the fragment ions with mass tolerance of 5 ppm.

## Figures and Tables

**Table 1:** Summary of *m/z* values and assigned chemical identities of fragmentation products of protonated cystine.

Protonated Cystine (Theoretical *m/z*)	Observed *m/z* Values of Fragment Ions (*n* = 3) ^a^		Proposed Chemical Identity	Theoretical *m/z*	Mass Error (ppm)	Reported in Previous Studies [Reference] ^c^
	Mean	SEM				
Cystine (241.03113)	224.00430	0.00007	[M + H − NH_3_]^+^	224.00458	−1.2	CID [4,15,16], others [19]
	205.99370	0.00015	[M + H − NH_3_ − H_2_O]^+^	205.99401	−1.5	unreported
	195.02547	0.00006	[M + H − H_2_O − CO]^+^	195.02565	−0.9	CID [4,14,15,16]
	177.99903	0.00006	[M + H − H_2_O − CO − NH_3_]^+^	177.99910	−0.4	CID [14,15,16]
	153.99895	0.00007	[M + H − C_3_H_5_NO_2_]^+^	153.99910	−1.0	CID [4,15,16]
	151.98341	0.00005	[M + H − C_3_H_7_NO_2_]^+^	151.98345	−0.2	CID [4,15,16], others [16,20]
	136.97256	0.00006	[M + H − C_3_H_5_NO_2_ − NH_3_]^+^	136.97255	0.1	Unreported
	122.02697	0.00006	[M + H − C_3_H_5_NO_2_S]^+^	122.02703	−0.5	CID [4,14,15,16], others [16,20]
	120.01137	0.00006	[M + H − C_3_H_7_NO_2_S]^+^	120.01138	−0.1	CID [4,15,16], others [16,20]
	105.00035	0.00006	[M + H − C_3_H_5_NO_2_S − NH_3_]^+^	105.00048	−1.3	unreported
	92.01640	0.00005	[M + H − C_3_H7NO_2_S − CO]^+^	92.01646	−0.7	unreported
	88.03925	0.00004	[M + H − C_3_H_5_NO_2_S − H_2_S]^+^	88.03930	−0.6	others [16,20]
	86.98979	0.00005	[M + H − C_3_H_5_NO_2_S − NH_3_ − H_2_O]^+^	86.98989	−1.4	unreported
	76.02145	0.00003	[M + H − C_3_H_5_NO_2_S − H_2_O − CO]^+^	76.02155	−1.3	unreported
	**74.02359 ^b^**	0.00003	[M + H − C_3_H_7_NO_2_ − CH_2_S_2_]^+^	74.02365	−0.9	CID [4], others [16,20]
	**74.00582**	0.00003	[M + H − C_3_H_7_NO_2_S − H_2_O − CO]^+^	74.00590	−1.1	unreported
	58.99519	0.00002	[M + H − C_3_H_5_NO_2_S − NH_3_ − H_2_O − CO]^+^	58.99500	3.2	unreported

^a^ The mean and standard error of mean (SEM) of the *m/z* values were calculated using the data from three independent experiments performed on different days. ^b^ Isobaric fragment ions are shown in bold. ^c^ Collision-induced dissociation (CID): fragmentation products generated by CID (unit mass resolution) [4,14,15,16]; others: fragmentation products generated by other fragmentation techniques, including electron-induced dissociation (high resolution) [16], field desorption (unit mass resolution) [19], and laser microprobe (unit mass resolution) [20]; unreported: fragmentation products not reported in any previous studies.
